# Melatonin in Micro-Tom Tomato: Improved Drought Tolerance via the Regulation of the Photosynthetic Apparatus, Membrane Stability, Osmoprotectants, and Root System

**DOI:** 10.3390/life12111922

**Published:** 2022-11-18

**Authors:** Naveed Mushtaq, Shahid Iqbal, Faisal Hayat, Abdul Raziq, Asma Ayaz, Wajid Zaman

**Affiliations:** 1 Jiangsu Key Laboratory for Horticultural Crop Genetic Improvement, Nanjing 210014, China; 2 College of Horticulture, Nanjing Agricultural University, Nanjing 210095, China; 3 College of Horticulture, Zhongkai University of Agriculture and Engineering, Guangzhou 510225, China; 4 Institute of Crop Germplasm Resources (Institute of Biotechnology), Shandong Academy of Agricultural Sciences, Shandong Provincial Key Laboratory of Crop Genetic Improvement, Ecology and Physiology, Jinan 250100, China; 5 State Key Laboratory of Biocatalysis and Enzyme Engineering, School of Life Sciences, Hubei University, Wuhan 430062, China; 6 Department of Life Sciences, Yeungnam University, Gyeongsan 38541, Republic of Korea

**Keywords:** abiotic stress, drought, tomato, reactive oxygen species, oxidative stress, melatonin, photosynthesis, climate changes, antioxidant system

## Abstract

Environmental variations caused by global climate change significantly affect plant yield and productivity. Because water scarcity is one of the most significant risks to agriculture’s future, improving the performance of plants to cope with water stress is critical. Our research scrutinized the impact of melatonin application on the photosynthetic machinery, photosynthetic physiology, root system, osmoprotectant accumulation, and oxidative stress in tomato plants during drought. The results showed that melatonin-treated tomato plants had remarkably higher water levels, gas exchange activities, root system morphological parameters (average diameter, root activity, root forks, projected area, root crossings, root volume, root surface area, root length, root tips, and root numbers), osmoprotectant (proline, trehalose, fructose, sucrose, and GB) accumulation, and transcript levels of the photosynthetic genes SlPsb28, SlPetF, SlPsbP, SlPsbQ, SlPetE, and SlPsbW. In addition, melatonin effectively maintained the plants’ photosynthetic physiology. Moreover, melatonin treatment maintained the soluble protein content and antioxidant capacity during drought. Melatonin application also resulted in membrane stability, evidenced by less electrolyte leakage and lower H_2_O_2_, MDA, and O_2_^−^ levels in the drought-stress environment. Additionally, melatonin application enhanced the antioxidant defense enzymes and antioxidant-stress-resistance-related gene (SlCAT1, SlAPX, SlGR, SlDHAR, SlPOD, and SOD) transcript levels in plants. These outcomes imply that the impacts of melatonin treatment on improving drought resistance could be ascribed to the mitigation of photosynthetic function inhibition, the enhancement of the water status, and the alleviation of oxidative stress in tomato plants. Our study findings reveal new and incredible aspects of the response of melatonin-treated tomato plants to drought stress and provide a list of candidate targets for increasing plant tolerance to the drought-stress environment.

## 1. Introduction

Due to variations in the global climate, the drought frequency, intensity, and interval are currently rising and have reached alarming levels [1]. Drought stress is the most important element among all environmental aspects linked to the predicted consequences of climate change on worldwide plant production [2]. Drought stress is caused by below-average rain along with warmer climates, resulting in extensive plant damage and reduced yield. Though a lack of water is the direct source of drought stress, increased evapotranspiration due to a warming environment is assumed to be the primary reason for severe drying due to global climate change [3]. Water scarcity hinders plant osmotic regulation, resulting in turgor loss, decreasing cell division, damaging membranes, disrupting the energy balance, and lowering plant growth overall [4]. Drought stress also exacerbates leaf aging and, even for a short duration, causes critical yearly yield losses [5].

Melatonin is a low-weight compound that has been found in most living organisms. Initially, melatonin was recognized in the conarium, which regulates the circadian clock, immune system, behavior, fertility, and the activities of antioxidants [6]. Melatonin may also serve an essential primary function in plant development (germination, vegetative phase, flowering, and delayed aging) and diverse stress responses as a naturally occurring antioxidant [7]. Melatonin has also enhanced resistance to various stresses, such as dehydration, salt, heavy metals, and cold and ambient temperatures [8]. Melatonin’s effect on plant stress tolerance is due to its ability to improve the plant antioxidant defense system [9]. Drought stress leads to a decline in plant production mainly due to the impairment of photosynthetic efficiency [10]. Likewise, a decline in photosynthesis activity initiated due to drought stress can trigger a drop in the reaction center in the plant’s PSII [11]. These changes can severely impair the photosynthetic apparatus if the plant cannot remove the additional energy [12]. Certainly, plants’ captured energy could be used to initiate the photosynthesis process. Secondly, it could utilize the chlorophyll fluorescence process as a result of re-emitting. Thirdly, this additional energy could be dispersed in the form of heat from the plants. These functions happen in a competitive manner; thus, a relative rise in the productivity of one process will decrease the efficiency of the remaining two processes in the plant [13]. Consequently, the plant’s capacity to disperse the extra energy can be analyzed by determining the chlorophyll fluorescence, especially under stress.

Plant root systems are important for adapting and surviving in a drought-stress environment [14]. It was previously revealed that melatonin treatment improved the root system in Arabidopsis and maize plants [15,16]. Drought also causes a rapid increase in ROS, inducing photo-inhibition, membrane injury, and oxidative stress damage in tomato plants [10]. In drought, excessive ROS accumulation can decrease the activity of chloroplasts, decrease photochemical efficiency, and ultimately decrease the photosynthesis and growth of tomato plants [11]. Plants possess antioxidant resistance mechanisms that include enzymes, such as SOD, APX, GR, POD, DHAR, and CAT, and non-enzymatic components, such as glutathione and ascorbate, all of which function together to counterbalance ROS and protect cells from oxidative injury [17]. In addition to these antioxidant functions, the tomato plant’s tolerance to drought stress involves the accumulation of osmoprotectants such as proline, which help to maintain its osmotic balance. In this manner, the proline level can help to reduce water potential by allowing water movement into the cell interior, reducing the injury caused by excessive ions. Proline accumulation is triggered under water stress, retains higher water in cells, and protects against protein impairment [18]. Similarly, the induction of different osmoprotectants, such as GB and trehalose, is associated with drought stress resistance in plants. Trehalose and GB significantly maintain the membrane stability and water level and enhance the ROS scavenging process in plants, helping them survive under drought stress [19].

Climate change has quickly evolved into a climate concern, with unanticipated impacts on agricultural yield. Because water scarcity is one of the biggest concerns for plants and, importantly, the world population’s future, assessing and discovering the ability of plants to grow with a limited water supply is critical [3]. Tomato (*Solanum lycopersicum*) is one of the most important vegetables grown globally and often encounters lower water availability because of climate change. In the current era, drought is the prime hindrance to tomato production in various areas around the world [20]. Improving drought resistance and maintaining tomato plants under water-limited conditions is a high-priority research focus [2]. However, several studies have revealed that the application of melatonin could improve drought tolerance in some plant species [7,21]. There is certainly no research focusing on the melatonin effects governing drought tolerance in Micro-Tom plants through osmoprotectant accumulation, membrane stability, and the protection of the photosynthetic apparatus. Furthermore, our research also determined the primary function of melatonin application in photosynthetic apparatus protection by employing the advancing field of plant phenotyping along with photosynthetic gene expression. We also explored the important role of melatonin application by analyzing the root system, ROS accumulation, water level, membrane damage, osmoprotectant genes, soluble proteins, polyphenol oxidase, the activation of antioxidants, and antioxidant capacity. This research provides details concerning melatonin’s role in enhancing tomato drought tolerance. 

## 2. Materials and Methods 

Micro-Tom tomato seeds (Jiangsu Academy of Agricultural Sciences, China) were sown in vermiculate after sterilizing and washing with distilled water. After germination, the tomato plants were placed in a growth chamber under 600 µmol m^−2^ s^−1^ light, with a relative humidity of 70%, a temperature of 24 °C, and a 15 h day/9 h night photoperiod. After 12 days, the tomato plants were moved to pots (24 cm × 14 cm) and transferred to the greenhouse. The plants were watered daily with Hoagland’s nutrient solution with a pH of 5.8. After 24 days, 96 tomato plants were separated into two groups. (1) Half (48) of the tomato plants were treated with 100 µM melatonin solution, and (2) the other 48 plants were treated with water only. The melatonin application was stopped after ten days. Next, four treatments were applied to the tomato plants in our study: (1) plants well-watered during the entire duration of the experiment, (2) well-watered plants pretreated with 100 µM melatonin application, (3) drought-stress-treated tomato plants that received the full water requirement for 12 days, followed by 10 days of water withholding without melatonin application, and (4) 10 days of water withholding in plants pretreated with 100 µM melatonin application (80 mL per tomato plant). The melatonin pretreatment was applied six times, with a 2-day interval, followed by up to 10 days of water withholding. Each stress condition included three replicates, using eight tomato plants per replicate. All stress treatments lasted for ten days; as in pilot experiments, tomato plants showed wilting on the ninth day of drought stress. The leaf samples were collected on the 10th day to conduct various analyses, including qPCR and biochemical and physiological examinations. 

### 2.1. Total Antioxidant Capacity

The total antioxidant capacity (TAC) was estimated in tomato leaves using an analytical system (BioQuoChem). It employs a photochemiluminescence (PCL) approach, which enables the quantification of the antioxidant status and water-soluble antioxidant capacity (ACW). Extracts for estimating the TAC were made following a prior method, and quantification was performed as instructed by the protocol in [22].

### 2.2. Polyphenol Peroxidase, Protein, and ROS Fluorescence

The polyphenol peroxidase (PPO) activity was measured by dissolving the 2 mL crude sample in 4 mL of caffeic acid solution and 2 mL of potassium dihydrogen phosphate (KH_2_PO_4_) buffer. The samples were placed in the incubator at 32 °C for 15 min. In the final step, the PPO absorbance was measured with a spectrophotometer at 380 nm. In tomato leaves, soluble protein quantification was performed via the method described by Bradford [23]. For this method, the enzyme sample was mixed with 0.9 mL of water and 6 mL of brilliant blue G–250 solutions. The samples were placed on a shaker for 1 min, and the final absorbance was measured at 595 nm.

Tomato leaves were initially soaked in a PBS solution (0.02 mM) for at least 10–14 min. H_2_DCFDA (15 um) was added after removing the PBS solution. Then, the leaves were vacuumed for 20 min and placed on slides to estimate the ROS fluorescence under a confocal microscope (Germany).

### 2.3. Endogenous Melatonin Content

In tomato plants, melatonin was estimated with a procedure illustrated in a previous study [24]. Two-gram leaf samples were crushed in liquid nitrogen, and 3 mL of acetone was added. Next, the tubes were placed into a shaker for 35 min at 28 °C. The tubes were centrifuged at nearly 3500× *g* (TGL-19 Benchtop High-Speed Multi-Functional Centrifuge, Noki, Zhengzhou, China) at 4 °C. After centrifugation, 3 mL of water was added to the tubes, and the samples were set up for measuring melatonin via high-performance liquid chromatography (HPLC, 1290 LC, Agilent, Santa Clara, CA, USA) equipped with a mass spectrometer (6470 LC-MS/MS, Agilent, Santa Clara, CA, USA). 

### 2.4. H_2_O_2_, MDA, and O^2−^

The levels of H_2_O_2_ were measured in tomato plants with a method explained in previous studies [25]. For this method, the level of peroxide in the solution was estimated by matching the absorbance to the standard curve. To estimate the MDA level, leaf samples were mixed with a PBS solution and placed in a water bath at 90 °C [21]. After that, the samples were cooled down at room temperature and centrifuged at 9000× *g* for about 6 min. After centrifugation, the supernatant was dissolved in TBA solution and vortexed for 2 min. The final reading of the solution was measured at 530 nm to estimate the MDA level.

The O_2_^−^ level in the tomato plant was calculated via the consequent protocol described by prior research [26]. Leaf samples (0.4 g) were dissolved in 5 mL of PBS buffer in the first step. Then, the samples were moved to ice for 18 min and centrifuged at 15,000× *g* for about 20 min. A total of 0.4 mL of the resultant solution was mixed with 0.4 mL of Na-PB solution, and then NH_2_OH.HCl solution was added to the tubes. The samples were then transferred for incubation at 28 °C for 40 min. 

### 2.5. Osmoprotectants and Enzymatic Activities

Proline and GB contents were measured in tomato leaves according to the methodology described by previous studies [27]. Glucose, sucrose, and fructose contents were determined by following a previously described technique [28]. The enzymatic process was followed to measure the starch contents, as defined by earlier research [29]. Trehalose content was calculated in tomato leaves by employing the technique reported by prior research [30]. The proline enzyme SlP5CS and SlP5CR activities were analyzed by following the method illustrated by Rivero [31]. SlP5CS and SlP5CR activities were quantified at 630 nm [32]. SlBADH, SlSUS3, SlSPS, and SlT6PS activities were estimated via a previously illustrated approach [27].

### 2.6. Antioxidant Enzyme Activities

In tomato plants, the extraction of enzymes was performed through the procedure illustrated by [33]. After collecting leaf samples, the tissue was homogenized in cold PBS liquid. The samples were then centrifuged for 13 min at 7000× *g* and 5 °C. Furthermore, the enzyme activity was determined right away using the supernatant in the tubes.

The catalase (CAT) activity was finally measured by observing the reduction in absorbance of about 240 nm in the spectrophotometer [33]. The sample tubes contained 40µL of PBS liquid (pH 7.0) and 2 mL of 0.3% H_2_O_2_. Ninety microliters of enzyme extract was used in the tubes to start the process. The SOD analysis was performed using the protocol of previous studies [14]. First, 0.8 mL of plant extract was added to the tubes. Next, 2 mL of Na_2_CO_3_, 0.6 mL of NBT, and 0.2 mL of EDTA were added to the tubes. Then, 0.3 mL of NH_2_OH·HCl was added to initiate the reaction in the tubes. In the final step, the reading was measured at 555 nm for about 2 min. The activity of APX was determined using a previously described method [34]. This protocol used a spectrophotometer to analyze the decrease in absorbance at 280 nm, which occurred during 5 min of the ascorbate oxidation process. The sample tubes contained 48 mM PBS liquid, 0.52 mM ascorbic acid, and 98 µL of crude enzyme. The reaction immediately started after the addition of 0.13 mM H_2_O_2_.

To analyze the DHAR activity in tomato plants, 0.12 mM EDTA, 0.18 mM DHA, 48 mM HEPES, and 2.6 mM GSH buffer were added to 19 µL of leaf extract. The extinction coefficient of 14 mM^−1^.cm^−1^ was used to analyze activity by determining the increase in the reaction at about 260 nm in the spectrophotometer [22]. The GR level was determined in tomato plants through the oxidation of NADPH according to a previously illustrated method [35]. In this process, the sample tubes contained 0.6 mM EDTA, 0.4 mM GSSG, 0.26 Mm NADPH, 48 mM HEPES buffer, and 98 µL of leaf extract. The reaction process was initiated by adding NADPH to the sample tubes. In the final step, the GR level was measured at 330 nm using the spectrophotometer. The POD level was analyzed in tomato leaves according to the method used in previous investigations [36]. First, the leaves were digested in 4 mL of PBS liquid. The tubes were centrifuged for 8 min at 25,000× *g*, and this liquid was used as the enzyme extract to measure the POD level. Next, 2 mM H_2_O_2_, 2.5 mL of PBS liquid, 8 mM guaiacol, and 48 mL of enzyme extract were added to the tubes. Finally, the POD level was monitored at 460 nm.

### 2.7. Chlorophyll Fluorescence and Gas Exchange Parameters

The following chlorophyll fluorescence parameters of tomato leaves were analyzed with the chlorophyll fluorescence apparatus ((Imaging PAM). This instrument measured the maximum quantum yield of PSII (Fv/Fm), effective PSII quantum yield (PSII), non-photochemical quenching (NPQ), and the electron transport rate using the Imaging Win application. The tomato leaves were dark-acclimated for 40 min before estimating the above parameters [10]. The photosynthesis-related parameters were analyzed in all tomato plants after ten days of treatment. These analyses were performed on the fully opened 3rd leaf about 4 h after the beginning of the light treatment [10]. The parameters include the photosynthesis activity rate, transpiration, stomatal conductance, and CO_2_ assimilation with the use of a portable photosynthesis system (LI-COR Biosciences, Lincoln, NE, USA). 

### 2.8. Electrolyte Leakage and RWC

Electrolyte leakage was measured to determine membrane stability using a previous technique [2]. Tomato leaf samples (about 110 mg) from different plants were obtained for every treatment. Next, the leaf samples were washed with distilled water for about 10 min. The leaves were then incubated at ambient temperature for 28 h in test tubes with 20 mL of water. In these leaf tissues, the initial conductivity (C1) was determined. After that, the leaf samples were boiled for 90 min at the boiling temperature and placed at 25 °C to cool down. The conductivity of these samples was determined as C2. Finally, electrolyte leakage was analyzed as the percentage proportion of C1–C2 in tomato leaves. 

To calculate the fresh weight, fully extended leaves were taken after the stress treatments [27]. These leaves were then held for 14 h at a 23 °C temperature in water to determine the turgid weight (TW). After that, the leaves were placed at 85 °C for two days, and the dry weight (DW) was determined. Eventually, the relative water content (RWC) was calculated using the following method:

RWC = FW − DW/TW − DW × 100

### 2.9. The Root Activity, Morphology, and Fresh and Dry Weights

After 10 days of drought-stress treatment, three plant roots (per treatment) were taken, washed with water, and scanned via a root scanner to analyze the root parameters. The root activity was measured by following the triphenyl tetrazolium chloride method explained in previous research [37]. In a solution of 10 mL of PBS puffer and TTC (0.45%), the root samples (600 mg) were immersed. Afterwards, these samples were moved to a dark place for 4 h at 35 °C, and 2.2 mL of H_2_SO_4_ was added to the solution. Lastly, the roots were blended and moved to a solution of 12 mL of ethyl acetate; the solution absorbance was estimated at 485 nm. After the drought-stress phase, the root samples were also separated to estimate the fresh and dried weights. First, the root fresh weight was measured, and the roots were moved to different paper bags and placed in the oven to remove the moisture. The root dry weight was determined after drying samples at 100 °C for 20 min and 80 °C for two days.

### 2.10. RNA Extraction and qPCR Analysis

After 10 days of treatment, the second leaves of the plants were utilized for RNA extraction. For this purpose, the RNeasy Plant Mini Kit was used following the protocol guidelines. Furthermore, cDNAs were produced with the PrimeScript™ RT Reagent Kit (TaKaRa) as per the company’s directions. The primers used in this research are provided in Supplemental Table S1. For the qPCR analysis, the instructions of the SYBR Premix Ex Taq™ were followed, and the samples were placed in the Bio-Rad CFX-9 system. The transcript levels of genes were estimated by following the procedure of previous studies [27].

### 2.11. Statistical Analysis

All research parameter differences were distinguished by applying the ANOVA and LSD analysis program in the SPSS software (version 25.0). The research results are shown as the mean ± S.D. The heml program was utilized to create heat maps.

## 3. Results 

**Figure 1:**
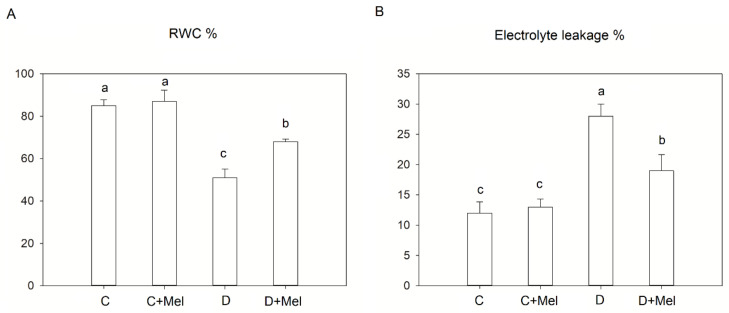
The physiological parameters relative water content (**A**) and electrolyte leakage (**B**) in tomato plant leaves under normal or 10-day drought-stress conditions with or without melatonin application. C: control; C+Mel: control with 100 µM melatonin pretreatment; D: drought, 10 days of withholding water; D+Mel: drought with 100 µM melatonin pretreatment. The values are the average of six replicates ± S.D. (*n* = 6). Significant differences among different treatments in the experiment were determined by LSD 0.05 test and are indicated by different letters.

To determine how the water status was affected in drought-stress environments, RWC was analyzed in plants. RWC revealed that melatonin-treated plants showed similar values to those found in untreated plants under well-watered conditions. RWC decreased in the drought-stressed plants compared to the control plants. Additionally, after 10 days of drought stress, the melatonin treatment maintained an elevated RWC compared to the untreated plants (Figure 1A). Furthermore, electrolyte leakage was also determined in tomato plants to evaluate the effect of drought stress on membrane stability. The melatonin-treated plants had the same electrolyte leakage values as the non-treated plants in a controlled environment. A significant increase in electrolyte leakage in the drought-stressed plants was observed compared to the control plants. Plants treated with melatonin revealed lower values of electrolyte leakage in comparison to the untreated plants in the drought environment (Figure 1B). These consequences suggest that the melatonin application supported membrane stability as an adaptation approach, which plays a primary role in increasing melatonin-treated plants’ drought resistance by retaining relatively high water content. 

**Figure 2:**
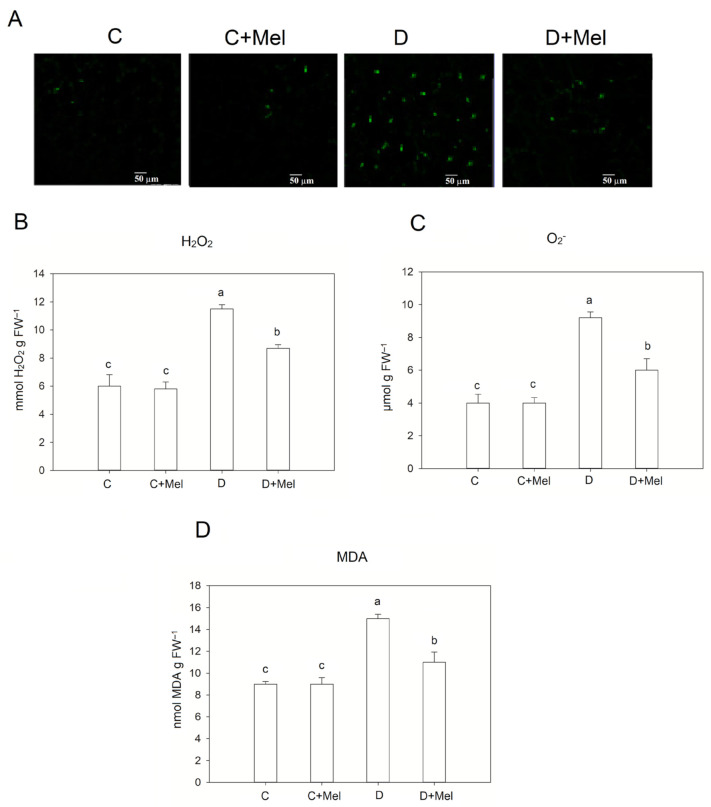
Impacts of melatonin application on the oxidative stress response in tomato plants. (**A**) ROS fluorescence and (**B**) H_2_O_2_, (**C**) O_2_^−^, and (**D**) MDA levels in tomato leaves after 10 days of drought stress. The green spots display the distribution of ROS. Bars, 50 µm. Data values are the means ± S.D. (*n* = 6). Significant differences among different treatments in the experiment were determined by LSD 0.05 test and are indicated by different letters.

Drought stress enhances the production of ROS, which impair the plant’s function and membrane stability. The fluorescence determination of ROS accumulation in leaf discs of tomato plants after ten days of drought stress revealed that ROS levels were lower in melatonin-treated plants (Figure 2A). Melatonin is highly effective for ROS removal and scavenging under environmental stresses [22]. 

H_2_O_2_ and O_2_^−^ levels were analyzed in tomato plants exposed to a water deficit. Under well-watered conditions, the application of melatonin showed no considerable change in the tomato plants. Under dry conditions, considerable H_2_O_2_ and O_2_^−^ accumulation occurred in the leaves; however, the melatonin-treated plants showed notably less H_2_O_2_ and O_2_^−^ (ROS levels) (Figure 2B,C). Additionally, the malondialdehyde (MDA) level was analyzed in plants to determine the protective effect of exogenous melatonin on membrane stability during drought conditions. Drought impaired the integrity of the cell membrane, as evidenced by elevated MDA levels in drought-stressed tomato plants (Figure 2D). However, in drought treatments, melatonin application significantly reduced MDA levels in plants.

**Figure 3:**
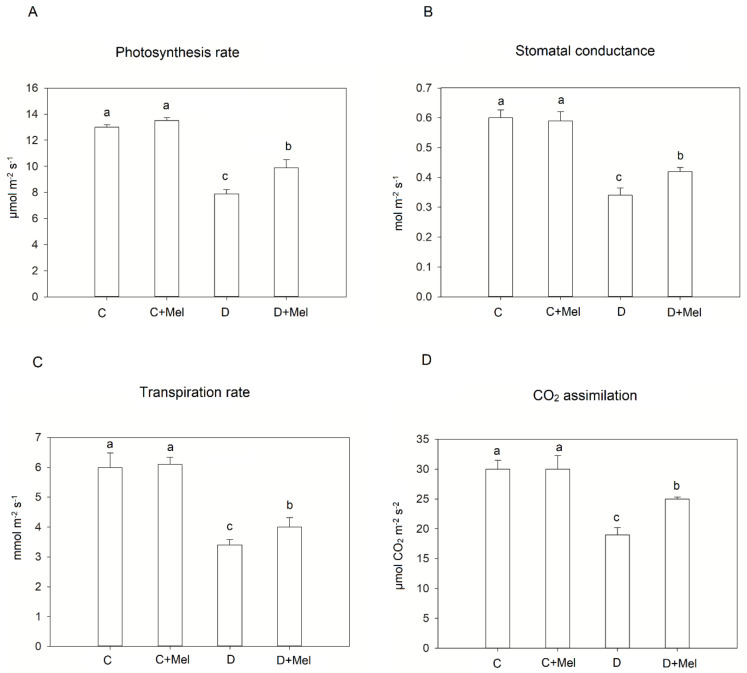
Melatonin application effect on tomato plants’ photosynthetic parameters: (**A**) photosynthetic rate, (**B**) stomatal conductance, (**C**) transpiration rate, and (**D**) CO_2_ assimilation rate after 10 days of drought stress. The values are the average of six replicates ± S.D. (*n* = 6). Significant differences among different treatments in the experiment were determined by LSD 0.05 test and are indicated by different letters.

The gas exchange parameters were assessed to investigate the effects of melatonin application on photosynthetic machinery in tomato plants. Under the control condition, melatonin had no evident effect on the gas exchange parameters, including the photosynthetic rate (Pn), transpiration rate (tr), CO_2_ assimilation (Ci), and stomatal conductance (St). The photosynthetic rate (Pn) and transpiration rate (Tr) decreased under the water-stress treatment compared to the control plants. The Ci and St parameters also showed the same decreasing trend under drought stress. We found that exogenous melatonin decreased these downtrends and improved the protective effect (Figure 3). These results confirm similar consequences, that is, that melatonin treatment protected the gas exchange parameters in plants under drought stress [9].

**Figure 4:**
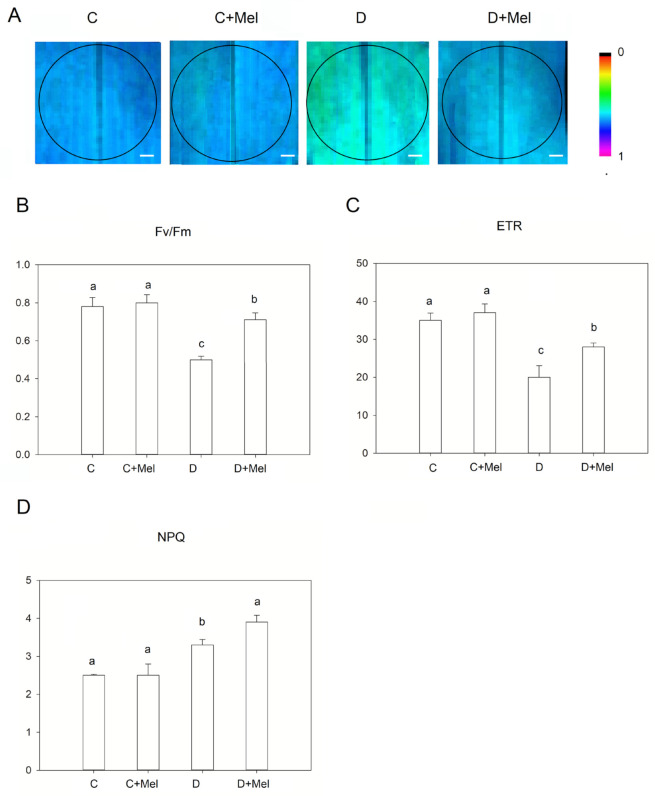
Chlorophyll fluorescence parameters in tomato plants after 10 days of drought stress. Fluorescence images of maximum PSII yield (Fv/Fm) (**A**), maximum PSII yield (Fv/Fm) values (**B**), electron transport rate (ETR) (**C**), and non-photochemical quenching (NPQ) (**D**). Scale bars represent 100 µm in the fluorescence images of maximum PSII yield. The values presented above are the average of six replicates ± S.D. (*n* = 6). Significant differences among different treatments in the experiment were determined by LSD 0.05 test and are indicated by different letters.

Chlorophyll fluorescence is an effective technique for determining the photosynthetic physiology of plants. Fv/Fm demonstrates the probable photosynthetic capacity of tomato plants, which illustrates the portion of captured photons consuming the photochemistry in the leaves (Figure 4A,B). Fv/Fm was not significantly affected by the melatonin treatment under control conditions. Ten days of drought stress considerably reduced Fv/Fm in the drought-treated plants compared to melatonin-treated plants. At the start of the drought-stress treatment, the ETR reading was similar to that of the control plants. The ETR decreased considerably in the drought condition after 10 days (Figure 4C). However, NPQ increased in the tomato plants after 10 days of drought stress (Figure 4D). Interestingly, melatonin-treated plants maintained higher ETR and NPQ than drought-treated plants. The above outcomes suggest that melatonin application might be involved in protecting Micro-Tom tomato plants’ photosynthetic physiology by maintaining normal PSII function and efficiency under drought stress.

**Figure 5:**
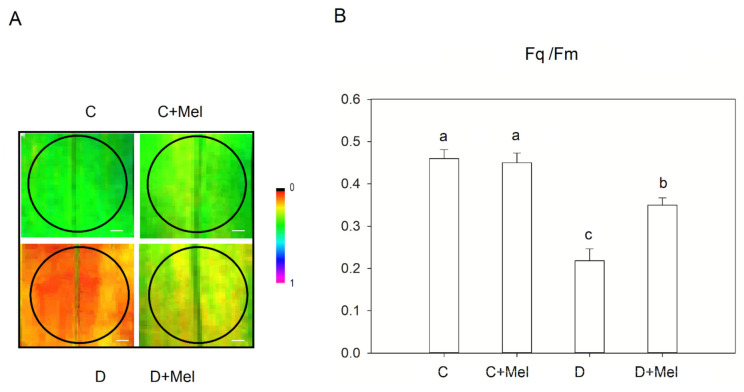
The effective efficiency of PSII (Fq/Fm) in tomato plants after 10 days of drought stress. Fluorescence images of (Fq/Fm) (**A**) and the values of (Fq/Fm) (**B**). Scale bars represent 100 µm in the effective efficiency of PSII. The values presented above are the average of six replicates ± S.D. (*n* = 6). Significant differences among different treatments in the experiment were determined by LSD 0.05 test and are indicated by different letters.

The effective efficiency of PSII (Fq/Fm) was analyzed to scrutinize the tomato plant’s photosynthetic effectiveness under drought stress (Figure 5). The black color represents a lower value, and the magenta color illustrates a higher value of Fq/Fm (Figure 5A). Our results showed that drought stress decreased the Fq/Fm values in tomato plants. Conversely, melatonin treatment ameliorated the negative effect of drought, implying that exogenous melatonin could inhibit the effective efficiency of PSII. Compared to the control plants, the melatonin-treated plants displayed no observable differences in the Fq/Fm values under well-watered conditions.

**Figure 6:**
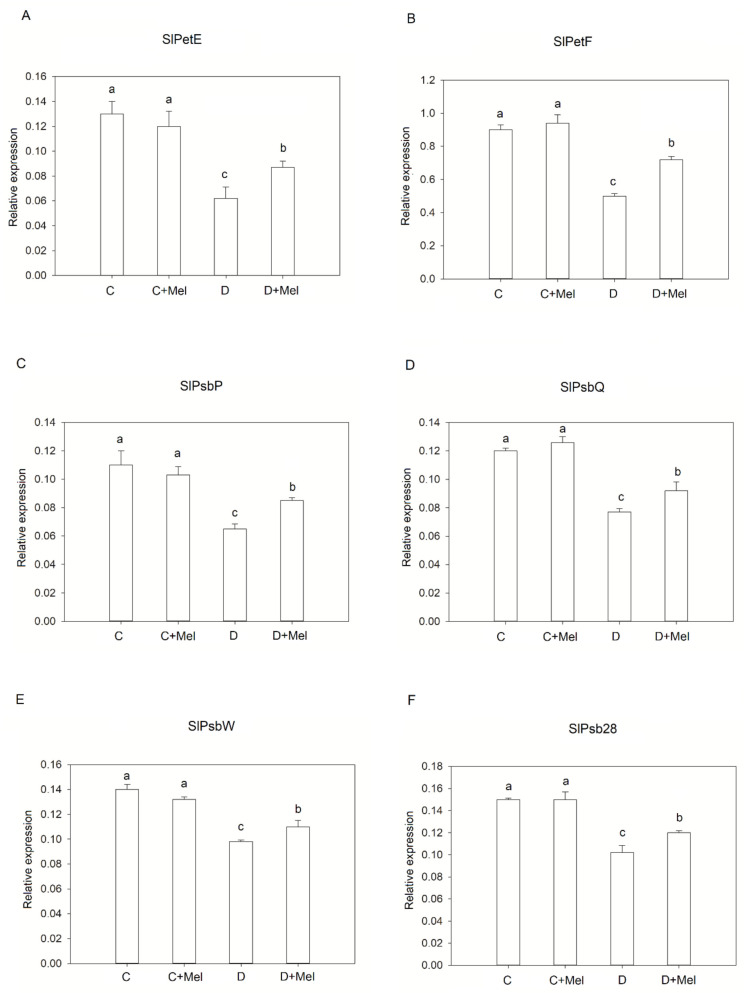
Effects of melatonin on the expression levels of photosynthetic-machinery-associated genes in tomato plants after drought stress. (**A**) SlPetE, (**B**) SlPetF, (**C**) SlPsbP, (**D**) SlPsbQ, (**E**) SlPsbW, and (**F**) SlPsb28 in melatonin-supplemented and non-supplemented plants under control or stress conditions. The values presented above are the average of six replicates ± S.D. (*n* = 6). Significant differences among different treatments in the experiment were determined by LSD 0.05 test and are indicated by different letters.

We examined the expression levels of several photosynthesis-related genes by qRT-PCR analysis during the stress treatment. Under control conditions, the expression of these genes was not different from that in the melatonin-treated plants. With the progression of water stress, the relative expression levels of photosynthetic genes were gradually downregulated. After 10 days of drought stress, the expression levels of SlPsb28, SlPetF, SlPsbP, SlPsbQ, SlPetE, and SlPsbW were significantly reduced compared to normal plants (Figure 6). We noticed that melatonin-treated plants exhibited high expression levels of these genes compared to drought-treated tomato plants. The results imply that melatonin application upregulates the expression of photosynthesis-related genes, thus retaining the PSII process in plants. 

**Figure 7:**
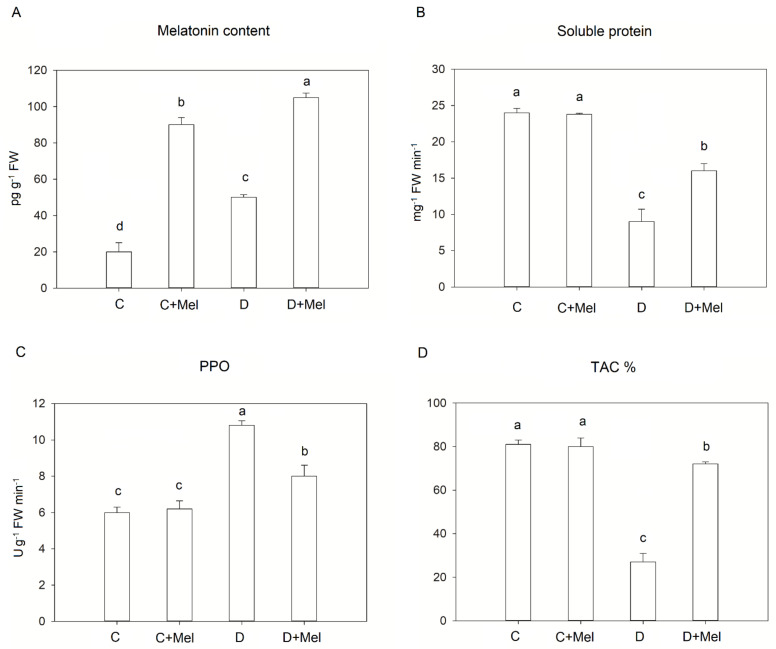
Changes in melatonin content (**A**), soluble proteins (**B**), PPO (**C**), and antioxidant capacity (**D**) in tomato leaves after melatonin treatment under 10 days of drought stress. The values presented above are the average of six replicates ± S.D. (*n* = 6). Significant differences among different treatments in the experiment were determined by LSD 0.05 test and are indicated by different letters.

The above studies show that melatonin is indeed synthesized and found in tomato plant leaves, and its endogenous level depends upon the stress conditions and their duration. Endogenous melatonin levels were measured in the leaves to determine the result of 10 days of drought stress on melatonin production (Figure 7A). In the control plants, the leaf’s melatonin content was about 19 pg/g^−1^ FW. The application of exogenous melatonin led to a significant difference in the melatonin level. Melatonin levels were significantly induced after plants were exposed to drought-stress conditions. Under drought stress, treatment with 100 µM melatonin increased endogenous melatonin levels by about 108 pg/g^−1^ FW. These findings reveal that melatonin is implicated in tomato plants’ response to drought stress. Exogenous melatonin treatment might change endogenous melatonin levels during stress to alleviate drought stress.

The tomato plants’ soluble protein contents were also measured under drought stress conditions. The soluble protein content was notably reduced in drought-stressed plants compared to well-watered plants. The melatonin application maintained the soluble protein content at higher levels than those in drought-treated plants (Figure 7B). Our outcome suggests that melatonin addition ameliorates the drought-stress effect through adjustments in PPO and soluble protein content in tomato plants. Drought stress effectively increased the activity of PPO in tomato plants, as perceived in Figure 7, compared to well-watered plants. The PPO activity decreased in melatonin-treated plants in comparison to drought-stressed plants. The melatonin-treated drought-stressed plants showed the most significant decrease in PPO activity compared to drought-treated plants (Figure 7C). 

In this study, the total antioxidant capacity (TAC) was also analyzed in tomato plants to determine the effects of melatonin application and its role in antioxidant system activation during drought stress. As tomato plants grew under well-watered conditions, their TAC values were the same as those of melatonin-treated plants. The TAC values were significantly reduced in the drought-treated plants compared to control tomato plants. Importantly, plants treated with melatonin exhibited higher TAC activity in comparison to drought-stressed plants (Figure 7D). This study indicates that melatonin application might significantly activate the TAC in tomato plants to eliminate ROS during drought stress.

Root parameters, including average diameter (AD), root activity (RA), root forks (RF), projected area (PA), root crossings (RC), root volume (RV), root surface area (RSA), root length (RL), root tips (RT), root numbers (RN), fresh root weight (FRW), and dry root weight (DRW), were analyzed to determine the effect of drought stress on tomato plants (Table 1). The current analysis indicated that the drought environment significantly affected tomato plants’ root-growth-related parameters. The results indicated that RA, RF, PA, RC, RSA, and RT were not improved by melatonin under control conditions. However, parameters such as RL, RN, FRW, RV, and DRW were significantly enhanced in melatonin-treated plants under control conditions. All root parameters were significantly decreased under drought-stress conditions, and, importantly, the application of melatonin alleviated these trends in melatonin-treated tomato plants.

**Figure 8:**
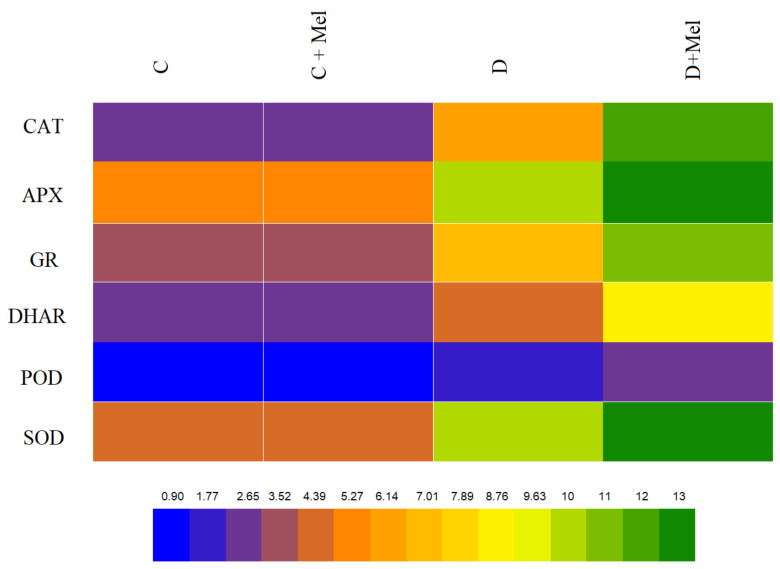
Effects of melatonin application on antioxidant defense enzyme activities. Catalase (CAT), ascorbate peroxidase (APX), glutathione reductase (GR), dehydroascorbate reductase (DHAR), peroxidase (POD), and superoxide dismutase (SOD) activities with or without melatonin in tomato leaves after 10 days of drought stress. The blue color illustrates lower values, and the green color illustrates higher values of antioxidant enzymes in the heat map. Significant differences among different treatments in the experiment were determined by LSD 0.05 test and are indicated by different letters.

Plants develop antioxidant resistance mechanisms to remove accumulated ROS and protect the plants from damage. Consequently, the activation of the antioxidants CAT, SOD, APX, DHAR POD, and GR in tomato plants with and without the application of melatonin was evaluated. The melatonin application did not change the activities of these six antioxidants under control conditions in tomato plants. The antioxidants CAT, SOD, APX, DHAR POD, and GR were induced under drought stress. Melatonin application further enhanced the activities of the antioxidants CAT, SOD, APX, DHAR POD, and GR compared to drought-treated plants (Figure 8). Our study indicates that melatonin application enhances the activation of the antioxidant defense system to maintain ROS in tomato plants.

**Figure 9:**
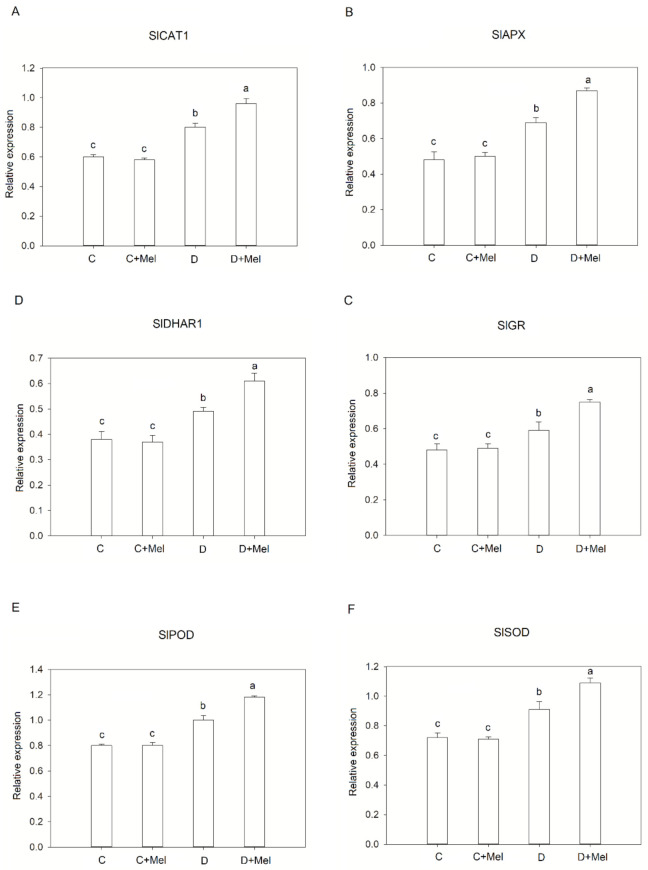
The expression levels of antioxidant-defense-related genes in tomato plants after 10 days of drought-stress treatment. SlCAT1 (**A**), SlAPX (**B**), SlGR (**C**), SlDHAR1 (**D**), SlPOD (**E**), and SOD (**F**) gene expression levels in tomato leaves with or without melatonin after 10 days of drought stress. The values presented above are the average of six replicates ± S.D. (*n* = 6). Significant differences among different treatments in the experiment were determined by LSD 0.05 test and are indicated by different letters.

The expression of transcripts related to the antioxidant pathway was measured under the drought-stress conditions employed in our study to fill some gaps in the understanding of the explicit role of melatonin in the activation of some stress-resistance-related genes (SlCAT1, SlAPX, SlGR, SlDHAR1, SlPOD, and SOD) in a climate change environment (Figure 8). The use of melatonin under well-watered conditions resulted in the same expression of the transcripts in comparison to control tomato plants. Drought stress significantly induced the transcript levels of these genes compared to well-watered plants. Tomato plants treated with melatonin showed the highest expression of antioxidant genes compared to drought-stressed plants (Figure 9).

**Figure 10:**
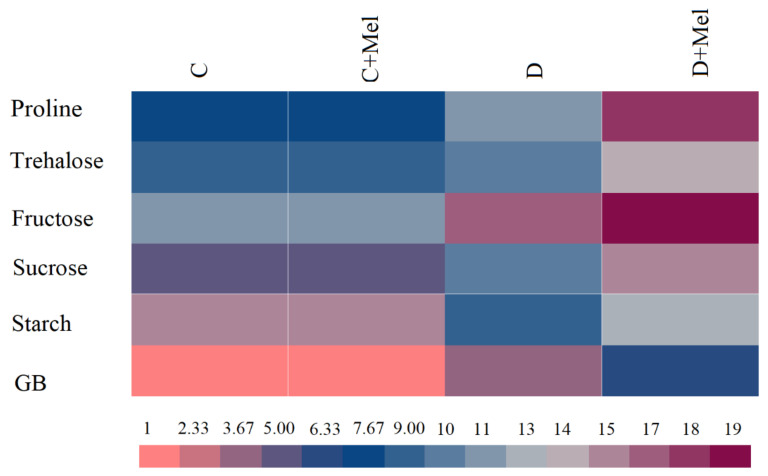
The activities of important osmoprotectants in tomato plants. Osmoprotectants (proline, trehalose, fructose, sucrose, starch, and GB) in melatonin-supplemented or non-supplemented tomato leaves after 10 days of drought stress. The pink color illustrates lower values, and the brown color illustrates higher values of osmoprotectants in the heat map. The values presented above are the average of six replicates ± S.D. (*n* = 6).

To investigate the osmoprotectant accumulation response to drought stress, we analyzed the accumulation of proline, trehalose, GB, glucose, sucrose, fructose, and starch in melatonin-treated and non-treated plants (Figure 10). The color range shows the osmoprotectant values, from pink (minimum) to brown (maximum). No apparent osmoprotectant accumulation differences were found after melatonin application in normal water conditions. After 10 days of drought, the accumulation of proline, trehalose, GB, glucose, sucrose, and fructose was perceived in the plants compared to normal plants. On the other hand, drought-treated plants had reduced starch content. Notably, melatonin-treated plants presented a higher concentration of these osmoprotectants than drought-treated plants.

**Figure 11:**
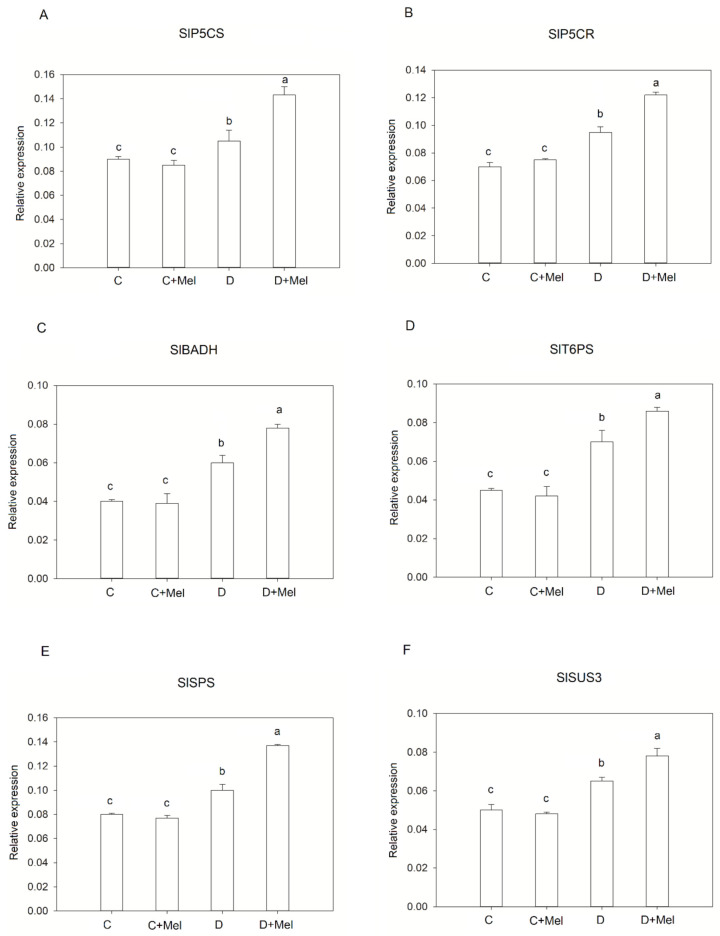
The expression levels of osmoprotectant genes in tomato plants. SlP5CS (**A**), SlP5CR (**B**), SlBADH (**C**), SlSPS (**D**), SlSUS3 (**E**), and SlT6PS (**F**) in tomato leaves with or without melatonin after 10 days of drought stress. The values presented above are the average of six replicates ± S.D. (*n* = 6). Significant differences among different treatments in the experiment were determined by LSD 0.05 test and are indicated by different letters.

The transcript levels of osmoprotectant genes were analyzed to determine the role of the osmoprotectant biosynthesis pathway in coping with drought-stress environments (Figure 11). In control conditions, melatonin application did not affect these genes’ transcript levels in tomato plants. Drought stress upregulated the expression of osmoprotectant genes compared to non-stressed tomato plants. Melatonin application further induced the expression of osmoprotectant genes such as SlP5CR, SlP5CS, SlSPS, SlBADH, SlT6PS, and SlSUS3 in tomato plants in the drought-stress phase. 

**Figure 12:**
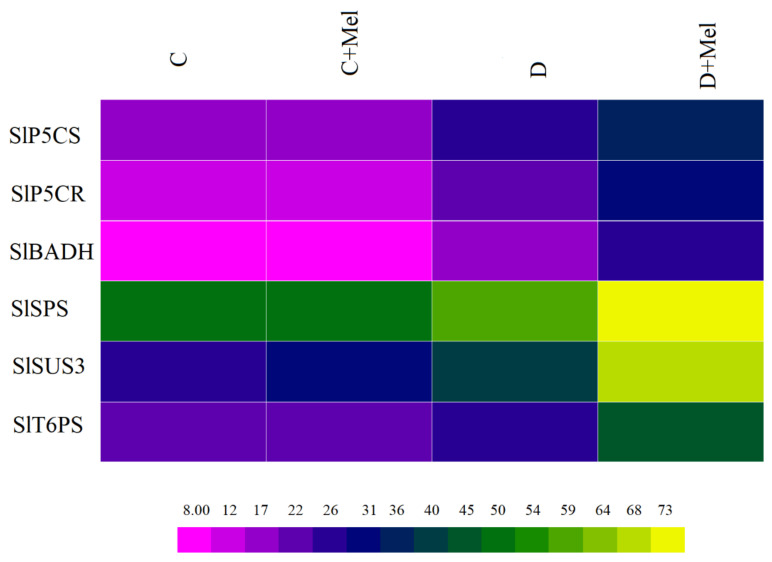
The enzymatic activity of osmoprotectants in tomato plants. SlP5CS, SlP5CS, SlBADH, SlSPS, SlSUS3, and SlT6PS were measured in melatonin-supplemented or non-supplemented tomato leaves after 10 days of drought stress. The magenta color illustrates lower values, and the yellow color illustrates higher values of osmoprotectants in the heat map. The values presented above are the average of six replicates ± S.D. (*n* = 6).

The osmoprotectants’ key enzyme activities were measured to further understand osmolyte accumulation in tomato plants. The color range shows the osmoprotectant values, from magenta (minimum) to brown (maximum). The accumulation of these enzymes was the same in melatonin-treated plants in well-watered conditions. The osmoprotectant enzymes were induced in drought-stressed plants compared to control plants. The application of melatonin significantly enhanced the activities of the osmoprotectant enzymes SlP5CR, SlP5CS, SlBADH, SlSPS, SlSUS3, and SlT6PS in the drought-stress environment (Figure 12).

## 4. Discussion

Dehydration stress critically restricts plant growth, decreases its water status, and reduces crop production. At the same time, melatonin treatment enhances plant resistance to dehydration stress by alleviating stress effects [8,24]. This study provides strong evidence that melatonin treatment improves drought tolerance in tomato plants by protecting the photosynthetic apparatus, photosynthetic physiology, membrane stability, and root system and enhancing the activity of the antioxidant defense system and osmoprotectants. Furthermore, melatonin-treated plants presented higher endogenous melatonin content under drought stress, implying that endogenous melatonin could be associated with drought tolerance.

RWC is one indicator of the plant water level, and its adjustment is associated with the adaptation to drought stress [10]. Drought stress significantly reduces RWC in tomato plants, resulting in decreased water movement from the roots to the stem, decreased mesophyll turgidity, lower leaf water potential, or a decline in soil moisture [38]. In our research, RWC was maintained in melatonin-treated tomato plants, which, overall, improved the performance of these plants due to membrane protection, as supported by prior research [16]. Melatonin has been shown to increase the cuticle’s thickness and ultimately prevent water loss through/from plants. Another study also demonstrated that melatonin treatment mediates tolerance to drought by maintaining the turgor and water ratio in plants [24].

MDA and electrolyte leakage are valuable indicators of cellular membrane damage induced by drought stress [33]. In the current research, drought-treated tomato plants showed a noticeable rise in electrolyte leakage. This outcome indicates that increased leakage could be caused by damage to membrane stability in plants due to 10 days of drought stress. Conversely, melatonin-treated plants showed decreased electrolyte leakage under the drought-stress scenario. In melatonin-treated plants, lower electrolyte leakage may be observed due to the protective mechanism’s activation. Our study is consistent with previous research showing that drought-tolerant plants displayed an increased membrane strength mechanism [39]. Our research also suggests that tolerance to drought stress might be associated with low lipid peroxidation levels in plants. Furthermore, drought stress causes ROS generation. This mainly consists of O_2_^−^ and H_2_O_2_, which function as signal transducers to activate cellular responses to stress [40]. The H_2_O_2_ and O^2−^ contents accumulated in melatonin-treated plants were considerably lower than those in plants not treated with melatonin. This outcome suggests that the enhanced drought resistance of plants could be due to effective ROS elimination. Our findings are highly consistent with previous research that demonstrated that lower H_2_O_2_ and O_2_^−^ contents are associated with tomato plants’ drought tolerance [41]. 

In plants, photosynthesis is the basis of plant growth and employs light energy to initiate the synthesis of organic compounds [10]. Drought is a major environmental stressor that prevents photosynthesis from taking place. The primary cause of the reduced photosynthetic rate during water stress is the limited ambient CO_2_ distribution, mediated by stomatal closure [42]. The maintenance of relative water content through melatonin could function in mediating gas exchanges and thus biochemical processes because melatonin-treated tomato plants had greater stomatal conductance, a higher photosynthetic rate, and increased transpiration, allowing a better source of assimilation for leaf tissues. The application of melatonin could also play a role in maintaining carboxylation efficacy in tomato plants under drought stress. Previous studies revealed that plants treated with melatonin maintained Pn, Tr, and Gs compared to untreated plants during drought stress [24,43].

To scrutinize the regulatory mechanism of melatonin for photosynthesis during drought stress, we investigated photosynthetic gene expression in plants. In our study, melatonin-treated plants showed higher gene expression of crucial photosynthetic genes than non-treated tomato plants in drought stress. Ferredoxin (Fd) and Plastocyanin (Pc) are essential components of the photosynthetic electron transport chain because of their crucial role in electron transfer [44]. Consequently, our research suggests that the high expression of these genes may have contributed to the improved electron transport rate and PSII efficiency in melatonin-treated plants in the drought-stress treatment. 

The use of chlorophyll fluorescence analysis to evaluate photosynthetic physiology in drought-stressed plants is a quick, sensitive, and non-invasive method [13]. The PSII photosynthetic machinery serves a critical function in the energy conversion process. Studies have shown that the drought-stress environment impairs the PSII reaction center in plants [12]. Our research suggests that melatonin treatment increases energy dissipation in tomato plants to improve the xanthophyll cycle. This process may increase the plant’s ability to endure drought stress and ultimately reduce PSII machinery damage in plants. The plants’ photosynthetic machinery functionality was observed under the drought-stress scenario by measuring Fv/Fm. Fv/Fm indicates the maximum photochemical efficacy of PSII and is utilized to reflect the level of injury to the photosynthetic apparatus in the drought environment [45]. In our study, Fv/Fm significantly decreased in non-melatonin-treated plants in the drought-stress environment. This could be due to the photosynthetic machinery being damaged. A previous study documented that melatonin can improve the efficiency of the electron transport rate in plants [46]. Similarly, the exogenous melatonin treatment efficiently improved the PSII reaction center response to drought stress in our study. This process maintained the electron transport rate and the efficiency of photochemical conversion in tolerant plants. Our research indicates that melatonin treatment may protect against the drought-induced impairment of the photosynthetic machinery. 

Polyphenol oxidase is the primary enzyme supporting plants’ defense system against stress by converting phenols to quinines, and PPO activity is induced under environmental stresses. A previous study revealed that plants under stress induce higher activities of PPO, which makes them more susceptible to these stress conditions [47]. In the current study, the PPO activity was significantly induced in drought-stressed plants compared to melatonin-treated plants. Our research outcomes are consistent with earlier research showing that melatonin application might alleviate oxidative stress damage in plants [48].

It is well recognized that the amount of soluble proteins in plants is a good indicator of their physiological status, especially under environmental stresses [49]. During water stress, the soluble protein content was considerably decreased compared to well-watered plants. Melatonin-treated drought-stressed plants maintained a higher soluble protein content than drought-treated plants. A previous study reported the same outcomes and proposed that drought stress decreased the soluble protein content by suppressing protein synthesis and triggering protein hydrolysis [50]. On the other hand, melatonin treatment may decrease protein breakdown while promoting new protein synthesis. Our studies suggest that exogenous melatonin may increase the generation of osmotic solutes, which play important roles in increasing plant-cell osmosis regulation and the water-holding capacity and controlling stomatal opening by removing ROS during drought stress.

Trehalose considerably improves and maintains the electron transport process in the PSII apparatus [27]. According to our findings, significant trehalose accumulation maintained the function of PSII in melatonin-treated plants. The maintained PSII function may be associated with the high electron transport rate, consistent with previous findings [51]. The plant’s ability to cope with drought stress depends on the effective regulation of electrons. Trehalose has been shown in several recent studies to be effective for protective mechanisms of the plant’s PSII machinery under stress conditions. Furthermore, treating buds with GB solutions significantly reduced H_2_O_2_ production, enhanced soluble sugar accumulation, and protected plant tissues from the impacts of environmental stress [52]. GB limits ROS formation and reduces lipid peroxidation in a stressful environment by protecting the photosynthetic machinery [53]. In our study, melatonin-treated plants significantly accumulated GB, the GB enzyme SlBADH, and key GB gene expression and decreased H_2_O_2_ and MDA levels after drought stress. This outcome suggests that GB could play an essential role in ROS scavenging and the activation of the antioxidant defense system to protect tomato plants.

Plants also initiate proline accumulation as a stress response to drought stress conditions. The activity of enzymes such as P5CR and P5CS is required to increase proline biosynthesis in plants [54]. Our research indicated that the proline level, the activity of the proline enzymes P5CR and P5CS, and the expression of the key proline genes SlP5CS and SlP5CR were induced in melatonin-treated plants. This outcome suggests that proline could have a role in stabilizing membrane structures, as evidenced by decreased electrolyte leakage in melatonin-treated plants, to cope with drought stress. In plants, high proline accumulation improves drought resistance [19,55]. A prior investigation indicated that the proline function stabilizes membranes and protein structures in plants [56]. Our research also observed significant sucrose accumulation in melatonin-treated plants under the drought-stress treatment. This process in melatonin-treated tomato plants might play a significant part in the increased energy observed in mitochondria under drought stress. Previous research showed that sucrose accumulation in plants protected cell mechanisms from the negative consequence of drought-stress treatment [57].

Climate-change-induced drought stress is frequently associated with elevated ROS levels in plants. Drought stress induces an increase in ROS generation, which affects plant growth, causes oxidative injury to the membrane, and, ultimately, plays a primary role in reducing plant efficiency in the drought-stress environment [10]. Plants have evolved a complex antioxidant defense system that includes non-enzymatic and enzymatic antioxidants to scavenge excess stress-generated ROS [17]. The important enzymatic antioxidants comprise CAT, GR, POD, APX, SOD, and DHAR, which perform crucial functions in protecting plants during drought stress [41]. In the current research, increased activities of APX, CAT, GR, POD SOD, and DHAR antioxidants was detected in melatonin-treated tomato plants under drought stress. This antioxidant defense system reduced drought-induced oxidative stress before it became harmful. Importantly, the potential of tomato plants to withstand 10 days of drought stress could be associated with the presence of the increased activity of these antioxidant enzymes, including POD, SOD, CAT, GR, and APX [58]. 

Root system enhancement regulates the ability of plants to attain nutrients and water. Root parameters directly affect plants’ capacity to absorb and transport available water and nutrients [59]. Improved root growth and length could effectively help withstand the damage caused by drought-stress conditions in plants. In addition, the root surface area and fresh and dry weights can be employed as critical indicators to estimate the drought tolerance of plants. Previous studies have shown that drought stress affects tomato plants’ root systems [60]. In our research, 10 days of drought-stress conditions affected the root system, including the average root diameter, root forks, projected area, root crossings, root volume, root surface area, root length, root tips, root numbers, fresh root weight, and dry root weight in Micro-Tom tomato plants. Nevertheless, tomato plants treated with melatonin showed protected roots in drought-stress scenarios. Previous studies reported that plants treated with melatonin enhanced their root system compared to untreated plants in the drought-stress phase [38,61]. Our study suggested that in Micro-Tom plants, melatonin treatment improved these plants’ root systems in particular, which indicates the stringent association between nutrient use and water withholding in drought stress. 

In the current research, a 100 µM melatonin treatment activated mechanisms that (1) maximally enhanced the ability to scavenge ROS in Micro-Tom tomato plants by initiating antioxidant metabolism and antioxidant defense genes, as well as by accumulating osmoprotectants that maintained the water status in leaves, which could lead to increased plant growth in the drought-stress phase. (2) The photosynthetic apparatus was protected to prevent ROS-induced oxidative stress damage by regulating photosynthetic activity, CO_2_ assimilation, maximum PSII yield, and non-photochemical quenching. (3) Melatonin treatment induced osmotic adjustment, maintained membrane stability, and retained the water balance, improving light energy absorption by electron transport in the PSII system. (4) The root system architecture was significantly enhanced, which could enable and improve access to water. Our study is in line with previous studies that illustrated that antioxidant metabolism, the protection of PSII, and the enhancement of the root system and membrane stability is a crucial feature of plants that helps them to survive in drought-stress environments [54,60,61]. 

## 5. Conclusions

Our research demonstrated that melatonin application helps alleviate the negative effects of drought stress in Micro-Tom tomato plants. Our study highlighted that melatonin application protected plants’ photosynthetic machinery and photosynthetic physiology. Furthermore, our study revealed the feasibility of treating Micro-Tom plants with exogenous melatonin, which improved the root system and decreased the sensitivity to drought stress. The ability of these tomato plants to increase the length, diameter, volume, and, importantly, the development of the root system is an imperative technological advance in horticulture crops. In addition, osmoprotectants and their related genes were evidently improved in melatonin-treated Micro-Tom tomato plants. In addition, melatonin-treated plants had less membrane damage after 10 days of drought stress, probably via the initiation of ROS scavenging by activating antioxidant enzymes and their related genes. This study unlocks a new research aspect for developing horticulture plants with enhanced tolerance to drought-stress environments. The overall intent is to minimize tomato plants’ yield losses in climate change environments.

## Figures and Tables

**Table 1:** Effect of melatonin treatment on the root parameters of Micro-Tom plants under drought stress.

	**C**	**C+Mel**	**D**	**D+Mel**
RN	35 ± 0.05 ^b^	40 ± 0.03 ^a^	16 ± 0.01 ^d^	26 ± 0.04 ^c^
RL (cm)	38 ± 0.02 ^b^	44 ± 0.07 ^a^	21 ± 0.06 ^d^	32 ± 0.03 ^c^
RV (cm^3^)	0.4 ± 0.001 ^b^	0.49 ± 0.002 ^a^	0.09 ± 0.003 ^d^	0.25 ± 0.006 ^c^
RSA (cm^2^)	45 ± 0.3 ^a^	46 ± 0.4 ^a^	10 ± 0.1 ^c^	22 ± 0.2 ^b^
RC	181 ± 0.9 ^a^	179 ± 0.6 ^a^	42 ± 0.4 ^c^	90 ± 0.8 ^b^
RT	699 ± 6 ^a^	696 ± 8 ^a^	189 ± 3 ^c^	380 ± 9 ^b^
RF	598 ± 4 ^a^	596 ± 9 ^a^	150 ± 2 ^c^	390 ± 5 ^b^
AD (mm)	0.5 ± 0.002 ^a^	0.47 ± 0.006 ^a^	0.12 ± 0.009 ^c^	0.35 ± 0.003 ^b^
PA (cm^2^)	13 ± 0.07 ^a^	12 ± 0.02 ^a^	4.2 ± 0.01 ^c^	7 ± 0.04 ^b^
RA (mg g^−1^ FW)	35 ± 0.05 ^a^	37 ± 0.09 ^a^	14 ± 0.02 ^c^	25 ± 0.01 ^b^
FRW (g)	2.7 ± 0.001 ^b^	3.2 ± 0.007 ^a^	0.5 ± 0.003 ^d^	1.52 ± 0.004 ^c^
DRW (g)	0.29 ± 0.002 ^b^	0.36 ± 0.001 ^a^	0.09 ± 0.06 ^d^	0.18 ± 0.05 ^c^

RN: root numbers; RL: root length; RSA: root surface area; RC: root crossings; RT: root tips; RF: root forks; AD: average diameter; PA: projected area; RA: root activity; FRW: fresh root weight; DRW: dry root weight. The values are the average of six replicates ± S.D (*n* = 6). Significant differences among different treatments in the experiment were determined by LSD 0.05 test and are indicated by different letters.

## Data Availability

Not applicable.

## Supplementary Materials

The following supporting information can be downloaded at https://www.mdpi.com/article/10.3390/life12111922/s1, Table S1: Primers used in this study.

## Abbreviations

APX, ascorbate peroxidase; KH_2_PO_4,_ potassium dihydrogen phosphate; PBS, phosphate-buffered saline; HEPES, (4-(2-hydroxyethyl)-1-piperazineethanesulfonic acid); CAT, catalase; DHAR, dehydroascorbate; NBT, nitro blue tetrazolium; EDTA, ethylene diamine tetra-acetic acid reductase; GC-MS, gas chromatography–mass spectrometry; HPLC, high-performance liquid chromatography; GR, glutatione reductase; MDA, malondialdehyde; PPO, polyphenol peroxidase; POD, peroxidase; qRT-PCR, quantitative real-time PCR; ROS, reactive oxygen species; RWC, relative water content; SOD, superoxide dismutase; TAC, total antioxidant capacity; Na_2_CO_3_, sodium carbonate; NH_2_OH·HCl, hydroxylamine hydrochloride; ACW, water-soluble antioxidant capacity; PCL, photochemiluminescence.

## References

[1] Liu D., Zhang C., Ogaya R., Fernández-Martínez M., Pugh T.A.M., Peñuelas J. (2021). Increasing climatic sensitivity of global grassland vegetation biomass and species diversity correlates with water availability. New Phytol..

[2] Mushtaq N., Wang Y., Fan J., Li Y., Ding J. (2022). Down-Regulation of Cytokinin Receptor Gene SlHK2 Improves Plant Tolerance to Drought, Heat, and Combined Stresses in Tomato. Plants.

[3] Zandalinas S.I., Balfagón D., Gómez-Cadenas A., Mittler R. (2022). Responses of plants to climate change: Metabolic changes during abiotic stress combination in plants. J. Exp. Bot..

[4] Yang X., Lu M., Wang Y., Wang Y., Liu Z., Chen S. (2021). Response mechanism of plants to drought stress. Horticulturae.

[5] Sperdouli I., Mellidou I., Moustakas M. (2021). Harnessing chlorophyll fluorescence for phenotyping analysis of wild and cultivated tomato for high photochemical efficiency under water deficit for climate change resilience. Climate.

[6] Kuwabara W.M.T., Gomes P.R.L., Andrade-Silva J., Júnior J.M.S., Amaral F.G., Cipolla-Neto J. (2022). Melatonin and its ubiquitous effects on cell function and survival: A review. Melatonin Res..

[7] Sun C., Liu L., Wang L., Li B., Jin C., Lin X. (2021). Melatonin: A master regulator of plant development and stress responses. J. Integr. Plant Biol..

[8] Khan M., Ali S., Manghwar H., Saqib S., Ullah F., Ayaz A., Zaman W. (2022). Melatonin function and crosstalk with other phytohormones under normal and stressful conditions. Genes.

[9] Hardeland R. (2016). Melatonin in plants–diversity of levels and multiplicity of functions. Front. Plant Sci..

[10] Liang G., Liu J., Zhang J., Guo J. (2020). Effects of drought stress on photosynthetic and physiological parameters of tomato. J. Am. Soc. Hortic. Sci..

[11] Yang X., Li Y., Chen H., Huang J., Zhang Y., Qi M., Liu Y., Li T. (2020). Photosynthetic response mechanism of soil salinity-induced cross-tolerance to subsequent drought stress in tomato plants. Plants.

[12] Hussain M.I., Reigosa M.J. (2011). Allelochemical stress inhibits growth, leaf water relations, PSII photochemistry, non-photochemical fluorescence quenching, and heat energy dissipation in three C3 perennial species. J. Exp. Bot..

[13] Harbinson J. (2013). Improving the accuracy of chlorophyll fluorescence measurements. Plant Cell Environ..

[14] Gapińska M., Skłodowska M., Gabara B. (2008). Effect of short-and long-term salinity on the activities of antioxidative enzymes and lipid peroxidation in tomato roots. Acta Physiol. Plant..

[15] Yang L., Sun Q., Wang Y., Chan Z. (2021). Global transcriptomic network of melatonin regulated root growth in Arabidopsis. Gene.

[16] Su X., Fan X., Shao R., Guo J., Wang Y., Yang J., Yang Q., Guo L. (2019). Physiological and iTRAQ-based proteomic analyses reveal that melatonin alleviates oxidative damage in maize leaves exposed to drought stress. Plant Physiol. Biochem..

[17] Hasanuzzaman M., Bhuyan M.B., Zulfiqar F., Raza A., Mohsin S.M., Mahmud J.A., Fujita M., Fotopoulos V. (2020). Reactive oxygen species and antioxidant defense in plants under abiotic stress: Revisiting the crucial role of a universal defense regulator. Antioxidants.

[18] Burritt D.J. (2012). Proline and the cryopreservation of plant tissues: Functions and practical applications. Current Frontiers in Cryopreservation.

[19] Semida W.M., Abdelkhalik A., Rady M.O.A., Marey R.A., Abd El-Mageed T.A. (2020). Exogenously applied proline enhances growth and productivity of drought stressed onion by improving photosynthetic efficiency, water use efficiency and up-regulating osmoprotectants. Sci. Hortic..

[20] Jangid K.K., Dwivedi P. (2016). Physiological responses of drought stress in tomato: A review. Int. J. Agric. Environ. Biotechnol..

[21] Li C., Tan D.-X., Liang D., Chang C., Jia D., Ma F. (2015). Melatonin mediates the regulation of ABA metabolism, free-radical scavenging, and stomatal behaviour in two Malus species under drought stress. J. Exp. Bot..

[22] Mestre T.C., Garcia-Sanchez F., Rubio F., Martinez V., Rivero R.M. (2012). Glutathione homeostasis as an important and novel factor controlling blossom-end rot development in calcium-deficient tomato fruits. J. Plant Physiol..

[23] Nicoli M.C., Elizalde B.E., Pitotti A., Lerici C.R. (1991). Effect of sugars and maillard reaction products on polyphenol oxidase and peroxidase activity in food. J. Food Biochem..

[24] Liu J., Wang W., Wang L., Sun Y. (2015). Exogenous melatonin improves seedling health index and drought tolerance in tomato. Plant Growth Regul..

[25] Brennan T., Frenkel C. (1977). Involvement of hydrogen peroxide in the regulation of senescence in pear. Plant Physiology.

[26] Elstner E.F., Heupel A. (1976). Inhibition of nitrite formation from hydroxylammoniumchloride: A simple assay for superoxide dismutase. Anal. Biochem..

[27] Rivero R.M., Mestre T.C., Mittler R.O.N., Rubio F., Garcia-Sanchez F., Martinez V. (2014). The combined effect of salinity and heat reveals a specific physiological, biochemical and molecular response in tomato plants. Plant Cell Environ..

[28] Blunden C.A., Wilson M.F. (1985). A specific method for the determination of soluble sugars in plant extracts using enzymatic analysis and its application to the sugar content of developing pear fruit buds. Anal. Biochem..

[29] Rasmussen T.S., Henry R.J. (1990). Starch determination in horticultural plant material by an enzymic-colorimetric procedure. J. Sci. Food Agric..

[30] Schulze U., Larsen M.E., Villadsen J. (1995). Determination of intracellular trehalose and glycogen in *Saccharomyces cerevisiae*. Anal. Biochem..

[31] Rivero R.M., Ruiz J.M., Romero L.M. (2004). Importance of N source on heat stress tolerance due to the accumulation of proline and quaternary ammonium compounds in tomato plants. Plant Biol..

[32] Geladopoulos T.P., Sotiroudis T.G., Evangelopoulos A.E. (1991). A malachite green colorimetric assay for protein phosphatase activity. Anal. Biochem..

[33] Camejo D., Jiménez A., Alarcón J.J., Torres W., Gómez J.M., Sevilla F. (2006). Changes in photosynthetic parameters and antioxidant activities following heat-shock treatment in tomato plants. Funct. Plant Biol..

[34] Nakano Y., Asada K. (1981). Hydrogen peroxide is scavenged by ascorbate-specific peroxidase in spinach chloroplasts. Plant Cell Physiol..

[35] Zafar S.A., Hameed A., Nawaz M.A., Ma W., Noor M.A., Hussain M., Mehboob ur R. (2018). Mechanisms and molecular approaches for heat tolerance in rice (*Oryza sativa* L.) under climate change scenario. J. Integr. Agric..

[36] Shah K., Kumar R.G., Verma S., Dubey R.S. (2001). Effect of cadmium on lipid peroxidation, superoxide anion generation and activities of antioxidant enzymes in growing rice seedlings. Plant Sci..

[37] Comas L.H., Eissenstat D.M., Lakso A.N. (2000). Assessing root death and root system dynamics in a study of grape canopy pruning. New Phytol..

[38] Altaf M.A., Shahid R., Ren M.-X., Naz S., Altaf M.M., Khan L.U., Tiwari R.K., Lal M.K., Shahid M.A., Kumar R. (2022). Melatonin Improves Drought Stress Tolerance of Tomato by Modulation Plant Growth, Root Architecture, Photosynthesis, and Antioxidant Defense System. Antioxidants.

[39] Farooq M., Wahid A., Kobayashi N., Fujita D., Basra S.M.A. (2009). Plant drought stress: Effects, mechanisms and management. Agron. Sustain. Dev..

[40] Rai G.K., Parveen A., Jamwal G., Basu U., Kumar R.R., Rai P.K., Sharma J.P., Alalawy A.I., Al-Duais M.A., Hossain M.A.J.A. (2021). Leaf Proteome Response to Drought Stress and Antioxidant Potential in Tomato (*Solanum lycopersicum* L.). Atmosphere.

[41] Ijaz R., Ejaz J., Gao S., Liu T., Imtiaz M., Ye Z., Wang T. (2017). Overexpression of annexin gene AnnSp2, enhances drought and salt tolerance through modulation of ABA synthesis and scavenging ROS in tomato. Sci. Rep..

[42] Liu D., Wu L., Naeem M.S., Liu H., Deng X., Xu L., Zhang F., Zhou W. (2013). 5-Aminolevulinic acid enhances photosynthetic gas exchange, chlorophyll fluorescence and antioxidant system in oilseed rape under drought stress. Acta Physiol. Plant..

[43] Nguyen T.T., Fuentes S., Marschner P. (2012). Effects of compost on water availability and gas exchange in tomato during drought and recovery. Plant Soil Environ..

[44] Zhang Y., Yu S.H.I., Gong H.-J., Zhao H.-L., Li H.-L., Hu Y.-H., Wang Y.-C. (2018). Beneficial effects of silicon on photosynthesis of tomato seedlings under water stress. J. Integr. Agric..

[45] Kusaba M., Ito H., Morita R., Iida S., Sato Y., Fujimoto M., Kawasaki S., Tanaka R., Hirochika H., Nishimura M. (2007). Rice NON-YELLOW COLORING1 is involved in light-harvesting complex II and grana degradation during leaf senescence. Plant Cell.

[46] Zhao H., Su T., Huo L., Wei H., Jiang Y., Xu L., Ma F. (2015). Unveiling the mechanism of melatonin impacts on maize seedling growth: Sugar metabolism as a case. J. Pineal Res..

[47] He F., Shi Y.-J., Zhao Q., Zhao K.-J., Cui X.-L., Chen L.-H., Yang H.-B., Zhang F., Mi J.-X., Huang J.-L. (2021). Genome-wide investigation and expression profiling of polyphenol oxidase (PPO) family genes uncover likely functions in organ development and stress responses in *Populus trichocarpa*. BMC Genom..

[48] Ahmad S., Wang G.-Y., Muhammad I., Chi Y.-X., Zeeshan M., Nasar J., Zhou X.-B. (2022). Interactive Effects of Melatonin and Nitrogen Improve Drought Tolerance of Maize Seedlings by Regulating Growth and Physiochemical Attributes. Antioxidants.

[49] Liang D., Ni Z., Xia H., Xie Y., Lv X., Wang J., Lin L., Deng Q., Luo X. (2019). Exogenous melatonin promotes biomass accumulation and photosynthesis of kiwifruit seedlings under drought stress. Sci. Hortic..

[50] Meng J.F., Xu T.F., Wang Z.Z., Fang Y.L., Xi Z.M., Zhang Z.W. (2014). The ameliorative effects of exogenous melatonin on grape cuttings under water-deficient stress: Antioxidant metabolites, leaf anatomy, and chloroplast morphology. J. Pineal Res..

[51] Luo Y., Wang W., Fan Y.Z., Gao Y.M., Wang D. (2018). Exogenously-supplied trehalose provides better protection for D1 protein in winter wheat under heat stress. Russ. J. Plant Physiol..

[52] Zhang G., Liu Y., Ni Y., Meng Z., Lu T., Li T. (2014). Exogenous calcium alleviates low night temperature stress on the photosynthetic apparatus of tomato leaves. PLoS ONE.

[53] Chen T.H.H., Murata N. (2008). Glycinebetaine: An effective protectant against abiotic stress in plants. Trends Plant Sci..

[54] Nounjan N., Nghia P.T., Theerakulpisut P. (2012). Exogenous proline and trehalose promote recovery of rice seedlings from salt-stress and differentially modulate antioxidant enzymes and expression of related genes. J. Plant Physiol..

[55] Montesinos-Pereira D., Barrameda-Medina Y., Romero L., Ruiz J.M., Sánchez-Rodríguez E. (2014). Genotype differences in the metabolism of proline and polyamines under moderate drought in tomato plants. Plant Biol..

[56] Evers D., Lefevre I., Legay S., Lamoureux D., Hausman J.-F., Rosales R.O.G., Marca L.R.T., Hoffmann L., Bonierbale M., Schafleitner R. (2010). Identification of drought-responsive compounds in potato through a combined transcriptomic and targeted metabolite approach. J. Exp. Bot..

[57] Jing F., Miao Y., Zhang P., Chen T., Liu Y., Ma J., Li M., Yang D. (2022). Characterization of TaSPP-5A gene associated with sucrose content in wheat (*Triticum aestivum* L.). BMC Plant Biol..

[58] Moustakas M. (2021). Plant Photochemistry, Reactive Oxygen Species, and Photoprotection. Photochem.

[59] Wang W., Ding G.-D., White P.J., Wang X.-H., Jin K.-M., Xu F.-S., Shi L. (2019). Mapping and cloning of quantitative trait loci for phosphorus efficiency in crops: Opportunities and challenges. Plant Soil.

[60] Xiong R., Liu S., Considine M.J., Siddique K.H.M., Lam H.M., Chen Y. (2021). Root system architecture, physiological and transcriptional traits of soybean (*Glycine max* L.) in response to water deficit: A review. Physiol. Plant..

[61] Tiwari R.K., Lal M.K., Kumar R., Chourasia K.N., Naga K.C., Kumar D., Das S.K., Zinta G. (2021). Mechanistic insights on melatonin-mediated drought stress mitigation in plants. Physiol. Plant..

