# Supplementary material for: Melatonin in Micro-Tom Tomato: Improved Drought Tolerance via the Regulation of the Photosynthetic Apparatus, Membrane Stability, Osmoprotectants, and Root System
Source: Life (Basel). 2022 Nov 18;12(11):1922. doi: 10.3390/life12111922 (PMC9696799; doi:10.3390/life12111922)
Supplement: Supplementary file 1 [file life-12-01922-s001.zip › life-2041655-supplementary.pdf]

Table S1: Primers used in this study

Gene	Forward	Reverse
SlActin	CTTGTCTGTGACAATGGAACTG	ATACCCACCATCACACCAGTAT
SlPetE	GTTCCCACACAACGTCGTA	GACAGTAACTTTGCCAACCA
SlPetF	TTCCTCACTCACAATGGCAAC	CCAGCTCTGTAGTTTTACCTT
SlPsbW	GGCCCTCCTACATAAGGCATC	GCCCAGTTCCTTCTGTACTCA
SlPsb28	GATTGTGCCTATCCCGTTC	ATCAAACCTGAATATAGCCAT
SlBADH	GCTTGGTGGAAAAAGTCCAA	AAGCAGCCAAAAAGAGTCCA
SlSUS3	TTGGATTTTGAGCCCTTCAC	AGAGAGGTGCCTGTTGAGGA
SlPOD	GGCCAATCTTTGACCCTTTA	AGTCCAGGAGCAAGTCCAGT
SlDHAR1	AGGTGGCTCTTGGACACTTC	CTTCAGCCTTGGTTTTCTGG
SlGR1	TTGGTGGAACGTGTGTTCTT	TCTCATTCACTTCCCATCCA
SlAPX	TCTGAATTGGGATTTGCTGA	CGTCTAACGTAGCTGCCAAA
SlCAT1	TGATCGCGAGAAGATACCTG	CTTCCACGTTCATGGACAAC
SlSOD	AGAAAGCTGTTGCTGTCCTTA	CCAGGAGCAAGTCCAGTTATAC
SlP5CS	CCCACACCGATAGCATCATTAC	TCCAAAGCGGAATCCATCAC
SlSPS	GATGAGTGAGATGGGGGAGA	TCTTGGTCCAAAAGGAATGC
SlT6PS	TGACAAACAGCAGGCTCATC	CTTCTTGACTTGGGGCTTTG
SlPsbQ	CGCTTGAGTACTGTTAGCACCA	AATGGCAAACTGAAGTCCCT
SlPsbP	AAAATTATATCACTCCGTGTC	GTATCTGAGAAGTCATCAGC
SlP5CR	GGAGCTGGTAAAATGGCTGA	CTGATCCAGAATGGGCTGTT
